# Wars between microbes on roots and fruits

**DOI:** 10.12688/f1000research.10696.1

**Published:** 2017-03-27

**Authors:** Ben Lugtenberg, Daniel E. Rozen, Faina Kamilova

**Affiliations:** 1 Institute of Biology, Leiden University, Sylviusweg 72, 2333 BE Leiden, Netherlands; 2 Koppert Biological Systems, Veilingweg 14, PO Box 155, 2650 AD Berkel en Rodenrijs, Netherlands

**Keywords:** rhizosphere, volatile organic compounds, antibiotic resistance, competitive colonization, biocontrol, fusaric acid, postharvest control

## Abstract

Microbes in nature often live in unfavorable conditions. To survive, they have to occupy niches close to food sources and efficiently utilize nutrients that are often present in very low concentrations. Moreover, they have to possess an arsenal of attack and defense mechanisms against competing bacteria. In this review, we will discuss strategies used by microbes to compete with each other in the rhizosphere and on fruits, with a focus on mechanisms of inter- and intra-species antagonism. Special attention will be paid to the recently discovered roles of volatile organic compounds. Several microbes with proven capabilities in the art of warfare are being applied in products used for the biological control of plant diseases, including post-harvest control of fruits and vegetables.

## Introduction

Soil is very poor in nutrients for microbes. The number of bacteria present in fertile agricultural soil is estimated at 10 ^9^ to 10 ^10^ per gram of soil ^1^. The density of bacteria near plant roots is 10 to 1000 times higher. This phenomenon is called the rhizosphere effect. The rhizosphere, defined by Hiltner as the layer of soil influenced by the root ^2^, contains nutrients representing 5 to 21% of the carbon fixed by the plant ^1^. The major nutrients present in the root exudates of dicotyledonous plants are (in order of quantity) organic acids, sugars, and amino acids ^3, 4^.

Although the resource levels in the rhizosphere are higher than in bulk soil, it is important to bear in mind that the nutrient concentration in the rhizosphere is approximately 100-fold lower than that in the common laboratory media. This is one of the reasons why it is not always warranted to assume that laboratory results can simply be translated to rhizosphere conditions.

Low nutrient concentrations can lead to strong competition between co-existing microbes that are often mediated by the secretion of toxic compounds. In this review, we will focus on these wars of aggression in the rhizosphere and on harvested fruits and vegetables. An outstanding source for understanding the mechanisms used in these wars is literature on the microbiological control of plant diseases caused by bacteria and fungi. For reviews, see 1, 5– 9. We will discuss molecules and traits that play a role in these processes as well as emphasize the recently discovered roles of volatile organic compounds (VOCs). Because the literature on wars among microbes is very extensive, we have restricted ourselves to broad principles that are illustrated with examples. Also, because mechanisms used in wars between beneficial microbes and pathogens are best known in cases when a Gram-negative bacterium is the beneficial attacker, the focus in this review is on these cases.

## War in the rhizosphere

Bacteria and fungi that control diseases caused by other microbes have been isolated widely from the rhizosphere. Competition in this environment and in others is classically divided into two types. The first, resource competition, includes traits or behaviors involved in nutrient acquisition and assimilation ^10^. Among these are resource-specific transporters or metabolic pathways that permit rapid growth across a range of resource concentrations. The second type of competition is interference competition, also known as allelopathy. By this process, microbes actively inhibit one another via diverse means in order to gain increased access to resources or space ^10^. While resource competition typically occurs in response to abiotic factors, interference competition is used in the face of biotic threats from competitors as a form of either offense or defense ^11^.

### Niche colonization

***Root colonization: competition for nutrients and niches.*** A competitive root tip colonization assay was developed by Simons *et al.* ^12^. Microbiologically sterile seeds are coated with a mixture of cells of two different strains, and then each coated seed is placed in a sterile system with a plant nutrient solution and allowed to germinate. When the root is approximately 10 cm in length, the number of microbes at various locations along the root is counted. These numbers are highest at the root base and decline rapidly towards the root tip. The ratio of the two types of microbes at the root tip appears to be a sensitive way to determine which strain is the best competitive colonizer ^12^.

The ability for bacteria to occupy specific sites on the root is a major route to ensure close proximity to the high concentrations of secreted nutrients. Many genes and traits required for efficient competitive root colonization have been identified (reviewed in 13– 15). The best-understood ones are the following: (i) chemotaxis towards root exudate compounds, where L-isoleucine was identified as the strongest chemoattractant in tomato root exudate but, when corrected for the quantities present, malic acid and citric acid are most effective ^16^; (ii) fast growth on root exudate components ^17^; and (iii) the type three secretion system, whose function involves pinching a needle through the plant cell membrane in order to extract nutrients from that source ^14^.

Chin-A-Woeng *et al.* ^18^ have shown that root colonization is essential for the control of the disease tomato foot and root rot (TFRR) caused by the fungal pathogen *Fusarium oxysporum* f. sp. *radicis-lycopersici* (Forl). This conclusion was based on the inability of three different competitive colonization mutants to efficiently colonize the root tip using the assay described above. Defective colonization was confirmed by experiments in soil.

The method of Simons *et al.* ^12^ was used by Kamilova *et al.* as a tool to enrich extremely efficient competitive root colonizers. They inoculated seeds with a mixture of total tomato rhizosphere bacteria. After root growth, they cut off the root tip, containing far less than 1% of the total number of bacteria present on the root. This root tip sample was intended to be enriched in bacterial strains that are superior competitors for exudate nutrients and for rapidly reaching the root tip. After two more enrichment cycles, whereby the root tip population was serially reinoculated via sterile seeds, individual root tip bacteria were purified and investigated. Most isolates were pseudomonads, and approximately half were superior in competition experiments for reaching the root tip niche after application on a sterile seed. In competition growth experiments in sterile exudate, the enhanced colonizers were at least 20-fold more effective than the best bacteria randomly selected from the starting mixture. Most importantly, four of the five tested enhanced colonizers appeared to control TFRR. So, it is clear that this method enriches for bacteria that exert biocontrol on roots via competitive exclusion. So far, this is the only class of biocontrol bacteria for which an enrichment method exists. This biocontrol mechanism was designated as competition for nutrient and niches (CNN) ^19^.

Another way to rapidly generate enhanced colonizers is to apply cells of a *mutY* mutator strain on sterile roots, followed by the enrichment procedure mentioned above. By this approach, rather than enriching for natural isolates, bacteria reaching the root tip have fixed a set of mutations that evolutionarily enhance their competitive colonization ability. The sets of mutations required for this increased colonization differ for the dicot tomato and the monocot grass ^20^; however, as yet, the individual effects of the fixed mutations remain uncharacterized.

***Colonization of fungal hyphae during biocontrol.*** Colonization of Forl hyphae has been illustrated for biocontrol strains *Pseudomonas fluorescens* WCS365 and *Pseudomonas chlororaphis* PCL1391 by confocal laser scanning microscopy ^21^. De Weert *et al.* ^22^ discovered that the bacterial cells move toward the hyphae by using fusaric acid (FA) secreted by the hyphae as the major chemoattractant.

**Figure 1.**
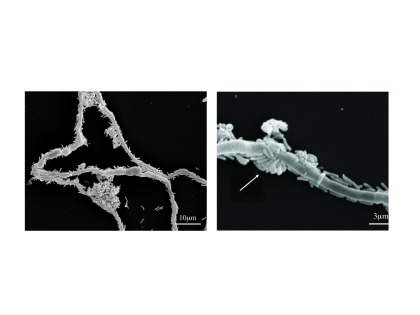
Scanning electron microscopy of colonization of *Fusarium oxysporum* f. sp. *radicis-lycopersici* (Forl) hyphae by cells of *Pseudomonas fluorescens* WCS365 after incubation for 72 hours in tomato root exudate. The right photo is a detail from the left photo but with a higher magnification (the arrow indicates a bacterial microcolony). Reproduced from Kamilova *et al.* ^23^ by permission of the publisher.

Kamilova *et al.* ^23^ observed that WCS365 cells colonize developing Forl hyphae and that enhanced colonization of the hyphae results in the formation of microcolonies ( Figure 1) and correlates with low nutrient availability. These results suggest that hyphal colonization is used to move to and occupy a high resource niche, e.g. fungal exudate. Colonization by the bacteria does not kill or deform the hyphae but results in slower hyphal growth. For further details on the effects of *P. fluorescens* WCS365 on Forl hyphae and spores, see also the section titled “Interference with activity, survival, germination, and sporulation of the pathogen” ^23^.

Hyphal colonization by other parasitic fungi has also been observed. Upon contact with fungi, the biocontrol fungus *Trichoderma harzianum* attaches to the host plant pathogenic fungus and can coil around it. This so-called hyphal mycoparasitism is suggested to be the mechanism by which *T. harzianum* controls *Sclerotinia sclerotiorum* in lettuce and sunflower seedlings ^24^.

### Molecular Weapons

The microbial arsenal is exceptionally diverse, with different weapons deployed in response to dangers of different magnitude and proximity. Thus, while the effects of secreted diffusible products like antibiotics, enzymes, siderophores, and bacteriocins are highly localized to the producing organism, the influence of volatile weapons is likely to be significantly broader ^25^. We briefly examine these differences below.

***Antibiotics.*** Examples of antibiotics used by Gram-negative biocontrol bacteria in the rhizosphere to attack fungal plant pathogens include 2,3-deepoxy-2,3-didehydro-rhizoxin ^26^, phenazine-1-carboxylic acid (PCA), phenazine-1-carboxamide (PCN), 2,4-diacetylphloroglucinol (Phl), pyrrolnitrin, 2-hexyl-5-propyl resorcinol, and the cyclic lipopeptide viscosinamide ^1, 6, 8, 9^ (for structures, see 27). It should be noted that strains FZB42 ^28^ and SQR9 ^29^ of the Gram-positive biocontrol bacterium *Bacillus amyloliquefaciens* are also sources of many antibiotics, such as fengycins, iturins, and surfactins ^28, 29^. Also, many fungi produce antibiotics, which can be active against bacterial and/or fungal plant root pathogens ^15, 30, 31^.

***Siderophores.*** Iron is an abundant element on the earth crust, but it is insoluble and therefore not suitable for uptake by living organisms. The free Fe ^3+^ concentration is only approximately 10 ^-18^ M. Microbes, like all organisms, need Fe ions. In order to scavenge Fe ^3+^ ions, they secrete siderophores, which are Fe ^3+^ ion-chelating molecules. Upon binding the ion, the formed siderophore–Fe ^3+ ^complex is bound by iron-limitation-dependent receptors at the bacterial cell surface, and the Fe ^3+^ ion is subsequently released and becomes active in the cytoplasm as Fe ^2+^ ^1, 15^. Examples of siderophores are pyocyanin and pyoluteorin (for structures, see 27).

The superior ability of certain bacteria to scavenge Fe ^3+^ ions can result in a reduction of the growth of pathogens. Examples of this form of biocontrol are the control of *Erwinia carotovora* (renamed as *Pectobacterium carotovorum)* by *P. fluorescens* strains ^32^ and the control of pathogenic fungi ^33, 34^. This form of biocontrol is most efficient when the Fe ^3+^ concentration is low, e.g. in acidic soils ^34^. An intriguing phenomenon is that similarities in siderophore structure can allow some species of bacteria to “pirate” those of others (called xenosiderophores), leading to iron deficits and growth or developmental arrest ^35– 37^. Since such xenopiracy also occurs within the rhizosphere, this offers interesting possibilities for biocontrol ^33, 34, 38^.

***Bacteriocins.*** Bacteriocins are structurally and functionally diverse antimicrobials that are generally distinguished from antibiotics by their narrow spectrum of activity against strains of the same species or congeners. Because of this narrow target range, bacteriocins are assumed to exert their strongest influence against direct competitors within the same ecological niche as the producing strain, a feature that makes them especially attractive for the purposes of biocontrol. Given the apparent ubiquity of bacteriocins – essentially all bacterial species carry a diverse set – we will only touch on this topic here, referring instead to several excellent reviews ^39– 41^.

Within the rhizosphere, pseudomonads produce strain-specific pyocins that vary in size and cellular target, designated R, S, and F-type bacteriocins. While S-type pyocins are related to structurally similar small peptide colicins from *Escherichia coli*, R- and F-type bacteriocins are evolutionarily derived from defective phages ^42^. In addition, pseudomonads including the well-known biocontrol strain *P. fluorescens* Pf-5 (renamed *Pseudomonas protegens*) produce lectin-like bacteriocins called putidacins or L-type bacteriocins, with genus-specific antimicrobial activity owing to the targeting of D-rhamnose within their cell walls ^43^. Importantly, putidacins have shown efficacy in field trials against *Pseudomonas syringae*, the causative agent of olive knot disease ^44^. Within soil and the rhizosphere, *Bacillus* bacteria also produce a vast array of bacteriocins with potential application to biocontrol. Notably, *Bacillus* bacteriocins seem to have broader target ranges, even extending to Gram-negative species ^45^. For example, a bacteriocin from *Bacillus subtilis* 14B is active against *Agrobacteria*, while another strain of the same species, IH7, reduces the incidence of damping off and seed-borne diseases when applied as a seed coating ^46, 47^.

Despite the promise of bacteriocins for biocontrol, it is important to bear in mind that the microbial targets of these agents are neither evolutionarily static nor defenseless. As with antibiotics, bacteria can readily evolve resistance to bacteriocins, thereby limiting their long-term efficacy. Equally, pathogenic bacteria produce many of the same bacteriocins as the biocontrol strains that are used to kill them. Indeed, bacteriocins are predicted to be one of the central factors driving bacterial diversification, an idea borne out by the tremendous diversity of bacteriocin loci within species ^48– 50^.

***Enzymes.*** Several soil-borne bacteria and fungi produce extracellular enzymes such as cellulases, chitinases, β-1-3 glucanases, lipases, and proteases. These lytic enzymes can hydrolyze a wide variety of polymeric fungal cell wall compounds, including cellulose, chitin, hemicellulose, and protein, thereby interfering with pathogen growth and/or activities. Production and secretion of these enzymes by different microbes can result in biocontrol. This mechanism, predation and parasitism, is a mechanism used by some biocontrol species of the fungus *Trichoderma* ^51^. Predation and parasitism is less well-known as a biocontrol mechanism used by bacteria, although a few examples exist ^52^. It should be noted that predation and parasitism is not always related to biocontrol. For example, the biocontrol strain *Collimonas fungivorans* is famous for eating live fungi, but its major mechanism of action for controlling TFRR is CNN ^53^.

***Interference with activity, survival, germination, and sporulation of the pathogen.*** Kamilova *et al.* ^23^ studied the interaction of WCS365 cells with Forl (see section titled “Colonization of fungal hyphae during biocontrol” and Figure 1) also in the presence of tomato root exudate. They observed that tomato root exudate stimulates fungal spore germination, an effect that can be mimicked by plant growth solution supplemented with citrate or glucose ^23^, the major organic acid and sugar compounds of exudate ^4^. However, the presence of WCS365 cells in exudate reduces spore germination and subsequent development of hyphae. Although the precise mechanisms remain uncertain, the authors suggest that the inhibition of spore germination by WCS365 is the result of consumption of nutrients that act as germination inducers ^23^. Furthermore, after the growth medium was limited in nutrients, abundant hyphae colonization by WCS365 cells was observed, supposedly as a result of their search for nutrients exuded by the fungus. Simultaneously, a reduction in microconidia production was observed. It therefore can be concluded that the reduction of spore germination, the colonization of developing hyphae ( Figure 1), and the subsequent inhibition of the production of new spores diminish fungal vigor as well as fungal dissemination, thereby contributing to the biocontrol of TFRR by WCS365 bacteria ^23^ whose major mechanism of biocontrol is induced systemic resistance (ISR) in the plant ^19^. See the following section for explanation of the latter mechanism.

***Indirect competition via induction of resistance in the plant.*** Interactions between selected plant growth-promoting bacteria and fungi with plant roots can prime these plants for resistance to a broad range of pathogens and insect herbivores while providing a competitive advantage for the inducing species. This biocontrol mechanism is called ISR and was recently reviewed by Pieterse *et al.* ^54^. The inducing microbes trigger a reaction in the plant roots that gives rise to a signal that spreads systemically throughout the plant and enhances the defensive capacity of distant tissues to subsequent infection by the pathogen. Examples of such microbes are species of the bacteria *Bacillus* and *Pseudomonas* and of the fungus *Trichoderma* and of mycorrhizal fungi. It should be noted that ISR differs from systemic acquired resistance, which is caused by a hypersensitive response triggered by plant pathogens.

In contrast to the situation with many other biocontrol mechanisms, extensive colonization of the root system is not required for ISR, as shown by experiments with *P. fluorescens* WCS365 ^55^ using root colonization mutants. Not only whole cells but also many individual bacterial determinants can induce ISR, such as lipopolysaccharide, flagella, salicylic acid, the siderophores pyochelin and pyocyanin, some cyclic lipopeptides, the antifungal factor Phl, the signal molecule acyl homoserine lactone (AHL), and volatile blends as well as the individual volatiles acetoin and 2,3-butanediol ^1, 27, 54^.

***Does malic acid of root exudates attract beneficial bacteria to the root?*** Rudrappa *et al.* ^52^ found that *Arabidopsis thaliana* seedlings whose leaves were infected with the foliar pathogen *P. syringae* pv. *tomato* DC3000 demonstrated enhanced root secretion of L-malic acid; in addition, they suggest that this elevated L-malic acid level selectively signaled and recruited the beneficial biocontrol rhizobacterium *B. subtilis* FB17, which defends the plant via ISR. Previously, De Weert *et al.* ^16^ reported that another biocontrol bacterium, *P. fluorescens* WCS365, which also defends the plant through ISR ^19^, shows strong chemotaxis toward the major tomato root exudate components, including malic acid. Although the suggestion of Rudrappa *et al.* ^52^ is intriguing, it seems unlikely that enhanced L-malic acid secretion can selectively attract beneficial bacteria given that chemotaxis to L-malic acid is found in both beneficial and potentially harmful bacteria.

***Volatile organic compounds.*** VOCs, complex mixtures of low-molecular-weight compounds, are produced by many organisms. Maffei *et al.* ^56^ proposed the name “volatilomes” for VOC mixtures. More than 1,000 bacterial VOCs have been described so far, but the diversity of environmental niches suggests that this is a gross underestimation ^57, 58^. Audrain *et al.* ^57^ divided bacterial VOCs into seven chemical classes. VOCs can travel far away from the site of production through the atmosphere, as well as through porous soils and liquids, making them ideal info-chemicals for mediating short- and long-distance interactions ^56^.

Microbial VOCs can exert a wide range of activities including controlling bacterial and fungal plant pathogens ^57, 59– 61^, signaling ^62^, inhibiting microbial activity ^57, 63^ and microbial growth ^57, 60, 64^, modifying drug resistance ^57, 63, 65, 66^, e.g. by raising the pH of the culture medium ^57^, negatively affecting biofilm formation ^57, 67^, eliciting ISR in a plant ^68– 73^, eliciting induced systemic tolerance to stresses caused by drought and heavy metals ^60, 69^, and promoting plant robustness ^74^ and plant growth ^68, 75^.

Excellent reviews about the chemical diversity and structures ^57, 76^ of VOC-producing microbes ^60, 76– 78^ and about the perspectives for application in sustainable agriculture and post-harvest control ^60, 79– 82^ have recently appeared.

VOC-mediated interactions between microorganisms can be easily demonstrated by using an I-plate, which is a Petri dish that contains two physically separated compartments that allows free exchange of air ^66^. However, the identification and quantification of VOCs requires special equipment ^57, 77, 83^. The volatile mixture can be analyzed using a combination of chemical profiles built by liquid chromatography–high resolution mass spectrometry or nuclear magnetic resonance and multivariate data analysis ^83^.

In the following paragraphs, we will discuss selected examples of activities of VOCs produced by microbes.

Volatilomes of many bacteria inhibit the mycelial growth of the plant pathogen *Rhizoctonia solani* ^77^. The strongest inhibition was found for volatiles of *Stenotrophomonas maltophilia* R3089, *Serratia plymuthica* HRO-C48, *Stenotrophomonas rhizophila* P69, *Serratia odorifera* 4Rx13, *Pseudomonas trivialis* 3Re2-7, *S. plymuthica* 3Re4-18, and *B. subtilis* B2g. Volatiles of strains of *P. fluorescens* and *Burkholderia cepacia* were moderately active. The VOC profiles of these antagonists differ in composition and complexity. Most volatiles are species specific, but overlapping volatile patterns were found for *Serratia* spp. and *Pseudomonas* spp. ^77^.

Using microarray analyses of *E. coli* exposed to the VOCs 2,3-butanedione and glyoxylic acid, Kim *et al.* ^63^ observed that these volatiles mediate global changes in gene expression related to motility and antibiotic resistance. They suggest that bacteria use airborne VOCs to sense other bacteria and to change master regulatory gene activity to adapt ^63^.

Raza *et al.* ^84^ reported that *P. fluorescens* WR-1, a biocontrol strain, produces *Ralstonia solanacearum* growth-inhibiting VOCs. Benzothiazole and 1-methyl naphthalene were the most effective at inhibiting growth, and the VOCs of *P. fluorescens* inhibited swarming, swimming, and chemotactic motility. These results suggest that the VOCs of the biocontrol strain, once established on the tomato root, not only reduce the growth of the pathogen but also inhibit movement of the pathogen towards the rhizosphere. Proteomics analysis showed 9 up-regulated and 19 down-regulated proteins. The latter include proteins involved in anti-oxidant activity, virulence, and carbohydrate and amino acid biosynthesis. The authors conclude that the results not only provide more insight into the mechanism of biocontrol but also could lead to the development of safe fumigants.

Many bacterial strains produce the antimicrobial VOCs ammonia and cyanide ^57, 58^. Biogenic ammonia modifies antibiotic resistance at a distance in physically separated bacteria ^65^. The cyanide ion is a potent inhibitor of many metalloenzymes, especially copper-containing cytochrome *c* oxidases ^85^. In addition to a role in the control of plant diseases ^6^, cyanide may play a role in increasing the availability of nutrients for plants through its ability to sequestrate metal ions ^86^. Dimethyl sulfide, produced by the rhizospheric bacteria *P. fluorescens* and *S. plymuthica*, has bacteriostatic effects on the plant pathogens *Agrobacterium tumefaciens* and *Agrobacterium vitis* ^87^.

One of the best-studied volatiles is 2,3-butanediol ^75^. This volatile elicits plant growth promotion ^75^ and ISR ^84^ in *A. thaliana*, as was shown using mutants blocked in the synthesis of 2,3-butanediol and its precursor acetoin ^88^. More recently, Ryu’s group studied rhizosphere colonization of pepper, using the wild-type 2,3-butanediol producer *B. subtilis* 168, its null mutant, and a 2,3-butanediol-overproducing derivative. The results showed that a higher level of 2,3-butanediol production correlates with improved rhizosphere competence of *B. subtilis* itself and that 2,3-butanediol production by *B. subtilis* suppresses the growth of the saprophytic fungus *Trichoderma* in the rhizosphere ^74^. Furthermore, growth experiments were carried out in exudates of roots pre-treated with the 2,3-butanediol-overproducing strain. This pre-treatment negatively affected the growth of the *B. subtilis* null mutant. This indicates that 2,3-butanediol protects *B. subtilis* (directly or indirectly) from harmful exudate components. The same pre-treatment also inhibited the growth of the soil-borne bacterial pathogen *R. solanacearum* but enhanced the growth of the saprophytic biocontrol bacterium *P. protegens* Pf-5. Apparently, 2,3-butanediol alters the root exudate composition ^74^.

The volatile 2,3-butanediol triggers strong ISR against necrotrophic bacteria but not against biotrophic ones. Park *et al.* ^89^ observed that the C16 volatile hexadecane confers protection against both types of pathogens. The C13 volatile tridecane, emitted by *Paenibacillus polymyxa*, was shown to be a more powerful inducer of ISR than was 2,3-butanediol ^70^.

### Defense strategies of pathogens

**Figure 2.**
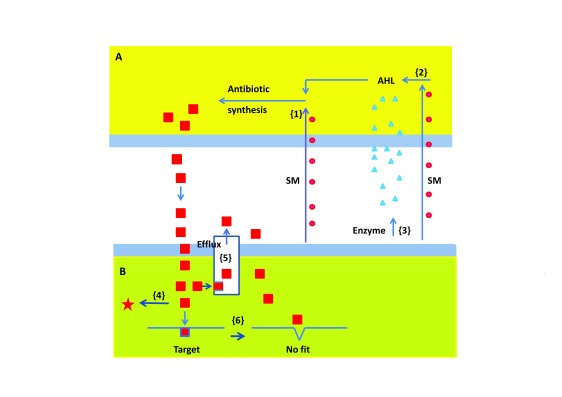
Major mechanisms used by microbes to defend themselves against antibiotic attack by other microbes. Microbe **A** (top) produces antibiotic molecules (red squares), which enter the antibiotic-sensitive Microbe **B** (bottom) and fit snugly in their molecular target, thereby inactivating Microbe **B** (see left part of figure). A variety of mechanisms can be used by Microbe **B** to defend itself. The numbers in brackets refer here, and in the figure, to the various defense mechanisms. {1} Microbe **B** can secrete a secondary metabolite (SM), which represses the expression of the antibiotic biosynthetic genes at the level of their promoter. In many cases, acyl homoserine lactones (AHLs) are required for activating antibiotic synthesis (see top right). In such a case, Microbe B can develop one of two different mechanisms. Firstly, it can secrete an SM which inhibits the synthesis of the AHL {2}. Secondly, Microbe B can secrete an enzyme which inactivates the AHL {3}. Also, after the antibiotic has entered Microbe **B**, **B** can develop various defense mechanisms. Firstly, it can produce an enzyme which inactivates the antibiotic {4}. Secondly, the antibiotic can be recognized by an efflux pump, by which it is secreted from the cell before it can do much harm {5}. Finally, the receiving microbe can alter its target in such a way that the antibiotic is not recognized any longer {6}. For further explanation, see the section titled “Defense strategies of pathogens”.

It is often assumed that resistance against biocontrol does not exist. Why this is not true is clearly explained in an excellent review by Duffy *et al.* ^90^. Here, we will summarize the known mechanisms of pathogen defense against biocontrol agents. They are illustrated in Figure 2, in which the numbers in brackets refer here, and in the figure, to the various defense mechanisms.

***Repression of weapon biosynthesis.*** The biocontrol strain *P. fluorescens* CHA0 produces Phl, which can kill the pathogen Forl. FA, a secondary metabolite secreted by Forl, appeared to repress Phl gene expression at the level of the *phlA* promoter ^91^ ( Figure 2 {1}).

Van Rij *et al.* ^92^ have shown that, in *P. chlororaphis* strain PCL1391, FA also represses the production of another antibiotic, PCN, but in a different way. Like in strain CHA0, antibiotic synthesis in PCL1391 requires activation of the quorum-sensing regulatory genes *phzR* and *phzI* by the AHL *N*-hexanoyl-L-homoserine lactone, which, like most AHLs, can diffuse freely through the membrane into the medium. The *phz* biosynthetic genes are activated only once the intracellular AHL level reaches a certain threshold concentration or quorum. This phenomenon is called quorum sensing. Van Rij *et al.* ^92^ discovered that FA in PCL1391 acts through inhibition of the synthesis of the AHL *N*-hexanoyl-L-homoserine lactone ( Figure 2 {2}). The reduction of the PCN level appears to be the result of direct or indirect suppression of the quorum-sensing regulatory genes *phzR* and *phzI* ^92^. This can be considered a form of signal interference (see the following section). Apparently, two different mechanisms have evolved for the inhibition of antibiotic synthesis in pseudomonads by Forl through FA secretion.

***Signal interference.*** Many bacteria produce antibiotics and other pathogenicity and virulence factors, such as fungal cell wall-degrading enzymes, only at a high bacterial cell density. The induction occurs when the level of quorum-sensing molecules such as AHLs reaches a certain threshold concentration in the growth medium (see 93, 94 and previous section).

Signal interference is a biocontrol mechanism based on the degradation of the AHL ^95^ ( Figure 2 {3}). Enzymes which are able to degrade AHLs are (i) AHL lactonases, e.g. of *Bacillus thuringiensis* strains, which hydrolyze the lactone ring ^95^, and (ii) AHL acylases, which break the amide link ^96^.

Interestingly, it was shown recently that AHLs also play a role in the formation of biofilms. AHL-degrading enzymes inhibit biofilm formation ^94, 96^, thereby preventing the microbes from becoming more tolerant to antibiotics.

***Detoxification of the weapon* ( Figure 2 {4}).** (i) Hydrogen cyanide (HCN) is produced by many biocontrol agents, especially pseudomonads. The cyanide ion is a potent inhibitor of many metalloenzymes, especially copper-containing cytochrome *c* oxidases. This volatile antibiotic shows little selectivity towards fungi ^6^. Osbourn ^97^ has reported that several fungi are able to detoxify HCN by degrading it to formamide.

(ii) Schouten *et al.* ^98^ screened a collection of 76 plant-pathogenic and 41 saprophytic *F. oxysporum* strains for sensitivity to Phl; 20 of these were relatively tolerant to high Phl concentrations, 18 of these were capable of metabolizing Phl, and, for two of these tolerant strains, it was shown that deacetylation of Phl to the less fungitoxic derivatives monoacetylphloroglucinol and phloroglucinol is (part of) the mechanism of Phl degradation.

***Active efflux of the weapon* ( Figure 2 {5}).** Schoonbeek *et al.* ^99^ reported that the expression of several ABC transporter genes of the fungal pathogen *Botrytis cinerea* is induced by the antibiotics Phl, PCA, and PCN. Phenazines strongly induce the expression of *BcatrB*. The *BcatrB* gene encodes one of the ABC transporters. *BcatrB* mutants are significantly more sensitive to phenazines than are their parental strain. Phenazine-producing strains of *Pseudomonas* are more strongly antagonistic *in vitro* to *BcatrB* mutants than to the parental *B. cinerea* strain. *Pseudomonas* strains that produce phenazines more effectively reduce gray mold symptoms induced by a *BcatrB* mutant than by the parental strain on tomato leaves. It can be concluded that it is likely that phenazines are secreted by the BcatrB protein ^99^.

***Modification of the target* ( Figure 2 {6}).** Osbourn *et al.* ^100^ observed that a range of pathogenic fungi can tolerate HCN and that, in some of these fungi, HCN tolerance is due to cyanide-resistant respiration.

***Dual role of fusaric acid in the war between the biocontrol bacterium P. chlororaphis strain PCL1391 and the fungal pathogen Fusarium oxysporum f. sp. radicis-lycopersici.*** One of the best-understood examples of attack and defense between microbes is the interaction between the fungus Forl and the bacterium *P. chlororaphis* strain PCL1391. The fungus secretes the secondary metabolite FA, which acts as a chemoattractant for the bacterium. This results in a high density of bacteria in the neighborhood of the fungal hyphae, in colonization of the hyphae, and eventually in biofilm formation on the hyphae. These high bacterial concentrations are ideal for quorum sensing and result in the production of the antibiotic PCN and subsequent killing of the fungus.

However, it appears that FA also has another role, namely in defending the fungus by inhibiting AHL production, and consequently also PCN production, by the bacterium. Based on this knowledge, one would predict that the bacterium can win only when a sufficiently high level of PCN is produced in a short period of time. This requires a high bacterial density. In practice, this can be reached, since *P. chlororaphis* strain PCL1391 is a good biocontrol bacterium ^18^.

***Is tolerance towards biocontrol agents an emerging threat?*** It is widely assumed, and often reported, that biocontrol strains use multiple mechanisms of attack. Since simultaneous tolerance development towards more than one mechanism in the same pathogen cell is unlikely, pathogen targets of biocontrol agents will suffer less from tolerance than organisms attacked by a single toxic compound. During the development of biocontrol agents, it would therefore be wise to select strains which use at least two mechanisms of activity against pathogens, also under the conditions where they will be used. Equally, it would be worthwhile to determine, in advance, the mechanisms of resistance to these agents and if these induce cross-resistance to diverse mechanisms of attack.

An alternative approach to reduce potential resistance would be to apply a combination of strains with different mechanisms of biocontrol. However, our experience with many such “cocktails” was that the biocontrol results were never better than that of a single strain and sometimes even worse (F. Kamilova and B. Lugtenberg, unpublished data). This negative result may be due to the limited “carrying capacity” of the root system for microbes. This would result in a dilution of the cell numbers of the beneficial strains on the root and therefore in a reduced biocontrol efficacy.

## Post-harvest control

Harvested fruits, vegetables, nuts, and grains contaminated with microbes have reduced shelf-life and quality and are less safe for human consumption. Products that are harvested and consumed fresh are often spoiled by fungal or bacterial rot and contaminated by food-borne pathogens ^79^. Certain microorganisms (e.g. antagonistic viruses, bacteria, yeasts, and multicellular fungi) can be implemented in the biological control of post-harvest problems. The very diverse mechanisms of action include CNN, antibiosis by antimicrobials and lytic enzymes, inhibitory volatile metabolites, pH decrease, parasitism, and induction of defense responses in the harvested plant product. Several mechanisms may act simultaneously ^79^.

Several commercial products containing strains of biological control agents are available as an alternative for, or as a complement to, chemicals that are traditionally used for post-harvest control. A list of relevant biological control agents used in post-harvest control is given in Table 21.2 in ^79^. Reported effective strains include bacteria belonging to species of *Bacillus*, *Pantoea*, *Pseudomonas,* and *Rahnella*, and fungal (including yeast) strains such as species of *Aureobasidium pullulans, Candida, Cryptococcus,* and *Metschnikowia*, and *Muscodor albus* ^79^.

An interesting example of post-harvest control by fungal volatiles (“mycofumigation”) was recently reported by the group of David Ezra ^101^. They observed that volatiles emitted by the fungus *Daldinia concentrica* prevent the development of mold fungi on organic dried fruits and eliminate *Aspergillus niger* infection in peanuts. They identified 27 VOCs and prepared mixtures that displayed strong activity against a wide range of fungi. In post-harvest experiments, these mixtures prevented the development of mold fungi on wheat grains and fully eliminated *A. niger* infection in peanuts. A mixture of 4-heptanone and *trans*-2-octenal was the most effective one, since it killed all the tested fungi. It is clear that *D. concentrica* and its volatiles are promising for use in the food industry and agriculture ^101^.

## Conclusions and future prospects

The mechanisms of wars between microbes described here are often based on studies in simple systems. Real horticultural and agricultural systems are much more complex and involve interactions of the discussed microbes with the whole microbiome, the plant, and the growth substrate.

Results of studies on war between microbes have already been applied in the microbiological control of plant diseases and of harvested fruits and vegetables. Microbial VOCs have great promise for further applications because they, or their derivatives, might replace harmful chemicals in agriculture and post-harvest control as biofumigants.

In the past decade, considerable progress has been made with the identification and activities of VOCs playing a role in the interactions between microbes. Of particular interest will be the elucidation of the influence of biotic and abiotic conditions, of the mechanisms of action of VOCs, and of the regulation of their synthesis. A start has been made with these studies. Results have shown that the volatilome is influenced by growth phase, substrate, oxygen concentration, moisture content, temperature, and pH (reviewed in 58, 102). Also, the presence of other microbes influences the volatilome ^102, 103^. At the intracellular level, the role of the Gac system in the regulation of the 205 VOCs produced by *P. fluorescens* SBW25 was investigated. It appeared that the synthesis of 24 VOCs is regulated by the Gac system ^104^. Eventually, all of these studies should shed light on the ecological role of VOCs.

Another interesting approach in elucidating communication between microbes is that of Liu *et al.* ^105^. They have carried out a set of intriguing experiments using a cucumber split root system to detect molecular signals in root exudate that mediate interactions in the rhizosphere. Their results, based on exudate analysis, correlation analysis, and testing pure compounds, indicate that pre-inoculation of one root side with the pathogenic fungus *F. oxysporum* f. sp. *cucumerinum* (Foc) results in enhanced tryptophan secretion by the roots at the other side as well as in enhanced colonization of the second root side by the biocontrol bacterium *B. amyloliquefaciens* SQR9. Similarly, pre-inoculation of one root side with SQR9 results in a reduction of raffinose secretion by the roots at the other side as well as in decreased colonization of the second root side by Foc. Tests with pure tryptophan and pure raffinose showed that tryptophan enhanced root colonization by SQR9 and that raffinose enhanced root colonization by Foc. It is clear that the colonization of roots with different microbes has different effects on the secretion of root exudate components. Future experiments along these lines are needed to test whether the plant indeed mediates wars between beneficial and pathogenic microbes by differentially influencing the exudation of molecular signals.

Recently, it was discovered that the type 6 secretion system (T6SS) can play a role in the colonization of both prokaryotic and eukaryotic competitors. The T6SS is a molecular machine used by a wide variety of Gram-negative bacteria to directly deliver toxins upon cell-to-cell contact into target cells in order to kill. The T6SS is a membrane-embedded, syringe-like system strikingly similar to the injection machinery of bacteriophages. The T6SS is supposed to be an evolutionary factor helping bacteria to conquer ecological niches ^106^. Ma *et al.* ^107^ have shown that the T6SS of *A. tumefaciens* deploys a superfamily of type VI secretion DNase effectors (Tde) as weapons for interbacterial competition *in planta*. These Tde inhibit antibacterial DNase activity. In an *in planta* co-infection assay, *A. tumefaciens* can use Tde to outcompete *Pseudomonas aeruginosa* after co-inoculation of both bacteria into tobacco leaves. Since the T6SS is widespread among Gram-negative bacteria, it may play an important role in root colonization as well.

Since microbial species have been shown to interact with each other to induce the production of active compounds ^11, 64, 103^, there currently is a trend in favor of the idea that, instead of applying single strains as active ingredients in plant protection products (PPPs), future PPPs should be based on a combination of species, preferentially with different mechanisms of action. Although this sounds like an intelligent improvement of the current practice, we see some hurdles. Firstly, as already mentioned in the section titled “Is tolerance towards biocontrol agents an emerging threat?”, plant roots have a limited carrying capacity to harbor microbes. Secondly, the costs of registration of PPPs based on a mixture of microbes would be much higher, as we will outline below.

PPPs based on several unregistered microorganisms with different modes of action would have a long and expensive route to the market because of registration rules, particularly in the EU but also in the USA. The active ingredient as well as the final (formulated) product needs to be evaluated. In case of a mixture, this means preparation of a dossier for each microorganism, which includes identification at the strain level, description of the biology of the microorganism, complex and expensive evaluation of its safety for humans and non-target organisms, and evaluation of residues and environmental fate. If we assume that the average costs of registration of a product based on a single microorganism varies between 300,000 and 800,000 Euros, the registration costs of a mixture of five strains would increase approximately five-fold. This cost becomes a serious financial burden for small- and medium-sized innovative biocontrol companies. Even in the case of a new product containing a mixture of already registered microorganisms, registration rules still require that the end product with the combination of strains be evaluated with respect to its physical and chemical properties and storage stability as well as its safety for humans and the environment. Also, the replacement of one strain of the mixture with a new strain or the addition of a new strain which was not registered previously would require repetition of the whole package of evaluation studies for the end product because it would be considered a new product. Finally, the idea of utilization of the whole microbiome as a PPP would presently not be possible, since current regulations do not envisage the application of unspecified microbiological mixtures as PPPs.

## Abbreviations

AHL, acyl homoserine lactone; CNN, competition for nutrient and niches; FA, fusaric acid; Foc, *Fusarium oxysporum* f. sp. *cucumerinum*; Forl, *Fusarium oxysporum* f. sp. *radicis-lycopersici*; ISR, induced systemic resistance; Phl, 2,4-diacetylphloroglucinol; PCA, phenazine-1-carboxylic acid; PCN, phenazine-1-carboxamide; PPP, plant protection product; Tde, type VI DNase effector; TFRR, tomato foot and root rot; T6SS, type 6 secretion system; VOC, volatile organic compound.
